# Concentrations of Metals in Tissues of Cockle *Anadara granosa* (Linnaeus, 1758) from East Java Coast, Indonesia, and Potential Risks to Human Health

**DOI:** 10.1155/2020/5345162

**Published:** 2020-01-06

**Authors:** Agoes Soegianto, Trisnadi Widyaleksono Catur Putranto, Wahyuhani Lutfi, Firdha Nur Almirani, Arfian Rahmat Hidayat, Andi Muhammad, Rachmadiva Aulia Firdaus, Yaniar Sari Rahmadhani, Desi Aina Nur Fadila, Dewi Hidayati

**Affiliations:** ^1^Department of Biology, Faculty of Science and Technology, Universitas Airlangga, Surabaya, Indonesia; ^2^Department of Biology, Faculty of Mathematics and Natural Sciences, Institut Teknologi Sepuluh Nopember, Surabaya, Indonesia

## Abstract

This study reports the presence of Cd, Pb, Zn, Hg, Cu, and Cr in the cockles (*Anadara granosa*, Linnaeus, 1758) harvested along the East Java Coast, Indonesia. The concentrations of metals were determined by atomic absorption spectrometer and expressed in mg kg^−1^ wet weight. The concentrations of metals ranged from 0.11 to 0.82 mg kg^−1^ for Cd, 0.10 to 0.54 mg kg^−1^ for Pb, 10.22 to 19.04 mg kg^−1^ for Zn, 0.02 to 1.47 mg kg^−1^ for Hg, 1.79 to 4.76 mg kg^−1^ for Cu, and 1.64 to 3.79 mg kg^−1^ for Cr. The metal concentrations in the whole tissues of cockles were in the order Zn>Cu>Cr>Hg>Cd>Pb. The Cd and Pb levels in cockles were found to be higher than the permissible limit for human consumption according to EC and FAO; the levels of Hg exceeded the EC, Hong Kong, Australia, and Indonesia standards; and the levels of Cr exceeded the Hong Kong standard. The estimated weekly intake (EWI) of cockles indicates that the concentrations of Cd and Hg in the cockle tissues from Gresik were higher than the provisional tolerable weekly intake (PTWI); meanwhile, the concentrations of Cr of cockles from all locations were higher than and close to the PTWI. The THQ values for Cd at Gresik, for Hg at Gresik, Surabaya, and Pasuruan, and for Cr at all locations were higher than one indicating that these metals pose potential noncarcinogenic effects to consumers. Reducing the consumption of cockles should be done in order to minimize the adverse effects of metals especially Cd, Hg, and Cr to human health.

## 1. Introduction

Recently, East Java Province of Indonesia has relatively fast economic growth, especially in the industrial sector. As a consequence of this rapid growth, significant impact of heavy metals could occur since heavy metals represent the major industrial contaminants of estuarine and coastal ecosystems. Aquatic organisms, including cockle in this ecosystem, can accumulate toxic metals which can pose a significant impact on human health due to the consumption of contaminated cockle. Heavy metals are nonbiodegradable toxic contaminants and may cause severe damage to the liver, kidney, central nervous system, mucus tissues, intestinal tract, and reproductive systems at high levels [1–3].

Cockle *Anadara granosa* (Linnaeus, 1758) is a typically intertidal species which naturally lives in an area with soft mud and fine sand. In some area, they can live in 20 m water depth but commonly concentrate in the littoral area and estuaries [4]. Cockles also live in estuaries and can tolerate some changes in salinity [5]. Cockles are shallow burrowers, filter feeders, and relatively sessile and feed on microscopic phytoplankton that are floating in the water column, as well as microphytobenthos in the sediment. However, both adults and juveniles are able to move to more favorable habitats when required [4]. *A. granosa* can be found in East Africa, Indo-West Pacific, Polynesia, Japan, and south to northern and eastern Australia [6].

Bivalve molluscs including cockles are relatively cheap sources of animal protein for people living in the coastal region of East Java. Gathering cockles from coastal beds is an important activity in the local fishing communities in East Java. Harvesting begins when the cockles have attained a marketable size of 25-30 mm. Thus, the contents of heavy metal in cockles collected along the East Java Coast are essential to be investigated, and potential health risk of heavy metal for coastal people trough seafood consumption should be evaluated.

Besides having an important economic value, bivalves have been well established as biomonitoring organisms to assess metal pollution due to their capability to accumulate metals within their tissues [7]. The use of bivalves as bioindicators for monitoring the concentration of heavy metals has been conducted in many areas in the world [8–16]. Due to their widespread distribution, cockles therefore can be used as biomonitoring organism for comparative study. The objectives of the present study were to measure the level of Cd, Pb, Zn, Hg, Cu, and Cr in the whole tissues of *A. granosa* collected from the coastal regions of East Java and to evaluate the potential health risk for human consumption by comparing with provisional tolerable weekly intake (PTWI) and target hazard quotient (THQ) guidelines.

## 2. Material and Methods

### 2.1. Sample Collection and Preparation

**Figure 1:**
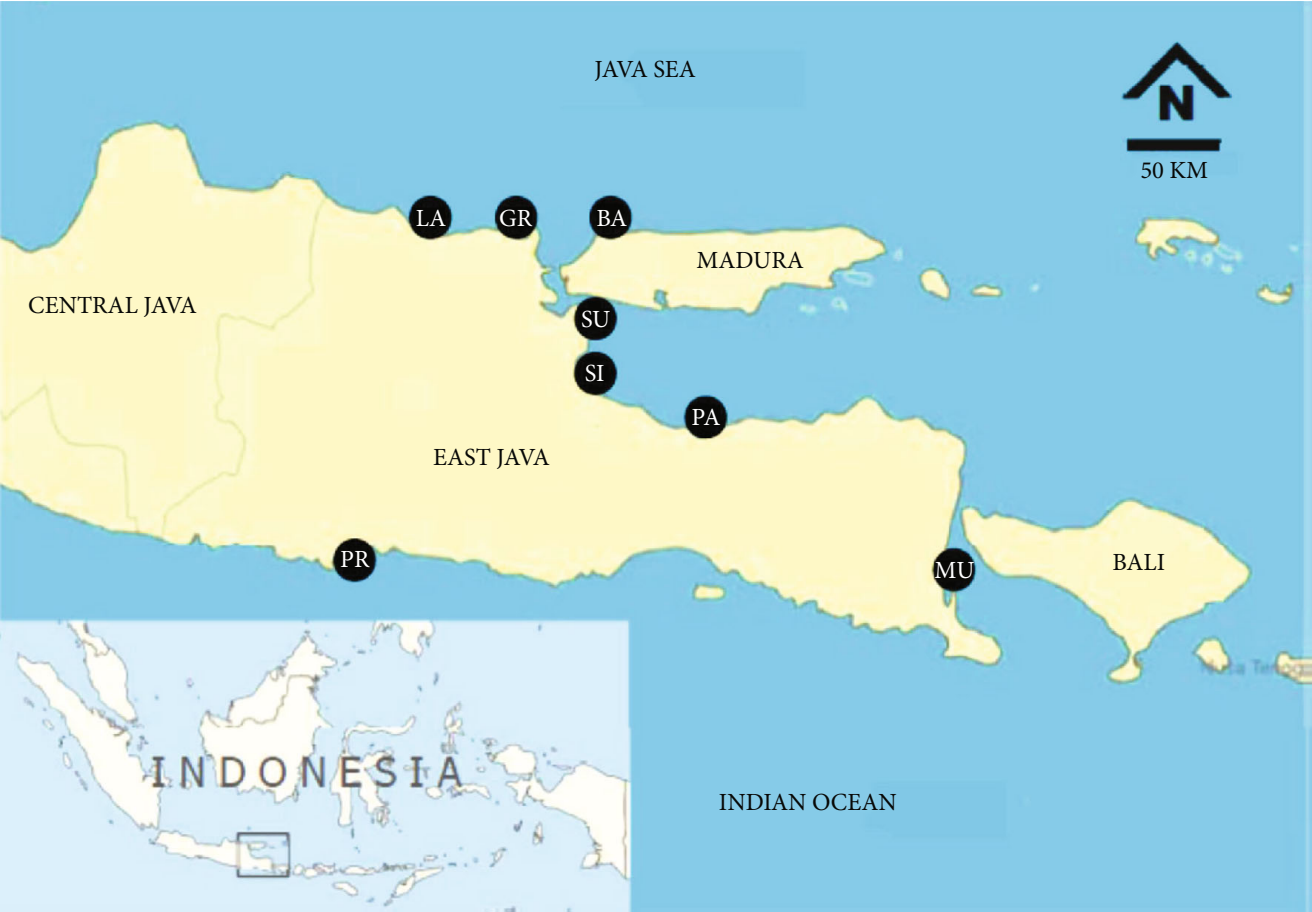
Sampling locations of cockle *Anadara granosa* at East Java Coast.

Cockles with edible size (3.5 ± 0.6 cm) were collected from eight selected sites of the East Java Coast, namely, Lamongan (LA), Gresik (GR), Bangkalan (BA), Surabaya (SU), Sidoarjo (SI), Pasuruan (PA), Muncar (MU), and Prigi (PR), during June to August 2018 and February to April 2019 (Figure 1). Those selected sampling sites were considered important cockle fishing areas in the East Java region.

After collection, cockles were rinsed with seawater at the time of sampling. The samples were then directly packed in a plastic bag, placed in a cool box, and brought to the laboratory on the same day. During transportation, the temperature of the ice box maintained near 4°C. In the laboratory, samples were stored in a freezer at -20°C for further analysis.

### 2.2. Measurement of Metals

Measurement of Cu, Zn, Cd, Pb, Cr, and Hg followed Candra et al. [17]. Whole tissues from approximately 30 cockles per sampling location were extracted from the cockle shells and then pooled to form a single sample. Six replications were applied for each location; therefore, each location required 180 cockle samples. The whole tissue was selected because it is consumed by local people.

Prior to metal analysis, external water from cockle tissue samples was absorbed using tissue papers; then, sufficient demineralized water was added to the pooled cockle tissues and put in a blender jar and homogenized at high speed for approximately three minutes. A subsample of homogenized cockle tissue from each location was dried at 60°C for 48 h until a constant weight was achieved. Approximately 2 g of dried tissue samples was thoroughly homogenized and digested using 5 mL concentrated HNO_3_ at 100°C for 3 h in microwave digester (Mars 6, CEM Corporation, North Carolina, USA). After cooling, samples were diluted to 50 mL with deionized water. An aliquot was taken for Cu, Zn, Cd, Cr, and Pb detection using flame atomic absorption spectrophotometer (ZEEnit 700, Analytik Jena AG, Jena, Germany). For measurement of total mercury (Hg), approximately 2 g of homogenized tissue sample from each sampling site was digested in acid solutions 2 mL of HNO_3_-HClO_4_ (1 : 1) and 5 mL H_2_SO_4_ at 80°C for 3 h in Mars 6 microwave digester. After cooling, 0.1 mL of KMnO_4_ and 0.5 mL of SnCl_2_ solutions were added until the purple color of the solutions stabilized. Sufficient hydroxylamine hydrochloride solution was added to neutralize the excess potassium permanganate as a preservative. The solution was adjusted to 50 mL with deionized water. An aliquot was taken for mercury determination using flameless atomic absorption spectrophotometer (Mercury-Hydride System Analytik Jena, HS 60). All metal concentrations of samples were expressed as mg kg^−1^ wet weight (ww). The metal concentration first determined as dry weight was transformed to the wet weight by dividing by a factor 4.856 (1 g dry weight ≈ 4.856 g wet weight). The detection limits of metals were as follows: Cu 0.04, Zn 0.07, Cd 0.01, Cr 0.01, Pb 0.01, and Hg 0.003 mg kg^−1^ ww.

Analytical blanks were run in the same way as the samples, and the concentrations were determined using standard solutions prepared in the same acid matrix. The accuracy and precision of the analytical performance were validated by measuring the dogfish muscle reference material (DORM-4) provided by the National Research Council of Canada. The recoveries for Cu, Zn, Cd, Cr, Pb, and Hg in the tissue standard reference material DORM-4 were 109, 94, 107, 93, 91, and 106%, respectively. All reagents used for this analysis were of analytical grade.

### 2.3. Estimation of Dietary Exposure to Heavy Metals

Estimated daily intakes (EDIs) of heavy metals for coastal people through seafood consumption were calculated using the following formula and is expressed as *μ*g per kg of body weight per day (*μ*g kg^−1^ BW day^−1^) [2, 18]:

(1) $$\begin{array}{c} \text{EDIs}=\left(\text{EF}\times \text{ED}\times \text{IR}\times C\right)/\left(\text{BW}\times \text{AT}\right) \end{array}$$

where EF is the exposure frequency (365 days year^−1^); ED is the exposure duration (70 years, equivalent to the average lifespan); IR is the ingestion rate of cockle (140 g person^−1^ day^−1^, as used in previous studies [16, 19]); *C* is the heavy metal concentration in cockle (*μ*g g^−1^ ww); BW is the body weight (60 kg for adults); and AT is the average time (it is equal to 365 days year^−1^ multiplied by the number of exposure years (70 years as assumed in this study)).

The estimation of weekly intake (EWI) of heavy metal equals to daily intake (EDI) multiply by seven days. The risks of dietary intakes of heavy metals were evaluated by comparing the weekly intake value with the provisional tolerable weekly intake (PTWI) according to the EC [20] for Cd and Pb, WHO [21] for Zn and Cu, WHO [22] for Hg, and USEPA [23] for Cr.

Target hazard quotient (THQ) was used to assess the noncarcinogenic health hazard for humans from each metal as a result of seafood consumption [24]. THQ was calculated using the following equation:

(2) $$\begin{array}{c} \text{THQ}=\text{EDI}/\text{RfD} \end{array}$$

where RfD (oral reference dose) values were obtained from the Integrated Risk Information System [24]. The RfD values for Cd, Cr, Cu, Zn, and Hg were 1, 3, 40, 300, and 0.3 (*μ*g kg^−1^ BW day^−1^), respectively [24]. The RfD value for Pb is actually under discussion [2, 3], so for its risk calculation, we used the value from Hang et al. [25], i.e., 3.5 *μ*g kg^−1^ BW day^−1^.

If the THQ value of heavy metal was less than one, it was assumed to not pose risk of noncarcinogenic effects (e.g., reproductive toxicity, teratogenicity, or liver toxicity) for humans over lifetime exposure. The estimates involved in these calculations present uncertainties; therefore, THQ values between one and ten may indicate a chance of noncarcinogenic effects, with an increasing probability for the occurrence of these effects as the value of THQ increases [2, 3, 25].

### 2.4. Statistical Analysis

All data were tested for fitness to a normal distribution by the Kolmogorov-Smirnov test. Because the data were normally distributed, differences in metal levels in cockles between sampling locations were examined using a one-way analysis of variance using SPSS Version 15.0. When significant differences were detected (*p* < 0.05), the Tukey post hoc test was used to determine which cockles were significantly different from different sampling locations.

## 3. Results

The concentrations of metals (Cd, Pb, Zn, Hg, Cu, and Cr) in the whole tissues of cockles collected from the East Java Coast are presented in Table 1. The levels of Cd in tissues of cockle varied from 0.11 to 0.82 mg kg^−1^, the highest Cd concentration was recorded in cockles from GR (*p* < 0.05), and the lowest was noted in cockles from PR. The Pb concentrations in cockles ranged from 0.10 to 0.54 mg kg^−1^. The highest Pb concentration was found in cockles from SU, and the lowest one was reported in cockles from MU (*p* < 0.05). The concentrations of Zn in cockles ranged from 10.22 to 19.04 mg kg^−1^. The highest Zn concentration was found in cockles from GR, and the lowest was reported in cockles from MU (*p* < 0.05). The concentrations of Hg in cockles ranged from 0.02 to 1.47 mg kg^−1^, with the highest Hg concentration found in cockles from GR (*p* < 0.05). The Cu concentrations varied from 1.79 to 4.76 mg kg^−1^, with the highest Cu level found in cockles from PA and the lowest Cu level noted in cockles from PR. The Cr concentrations ranged between 1.64 and 3.79 mg kg^−1^, with the highest level found in cockles from PA and the lowest level recorded in cockles from PR.

The estimated weekly intake (EWI) of metals by people from the Java Sea Coast ranged from 1.85 to 13.29 *μ*g kg^−1^ BW for Cd, from 1.68 to 8.74 *μ*g kg^−1^ BW for Pb, from 167.06 to 311.06 *μ*g kg^−1^ BW for Zn, from 0.34 to 24.06 *μ*g kg^−1^ BW for Hg, from 29.27 to 77.72 *μ*g kg^−1^ BW for Cu, and from 26.75 to 61.91 *μ*g kg^−1^ BW for Cr. (Table 2).

The THQs of selected metals were summarized in Table 3. The THQs of metals ranged from 0.26 to 1.91 for Cd, from 0.07 to 0.36 for Pb, from 0.08 to 0.15 for Zn, from 0.16 to 11.43 for Hg, from 0.10 to 0.26 for Cu, and from 1.28 to 2.78 for Cr. (Table 3).

## 4. Discussion

This study provided valuable information concerning the levels of metals in the tissue of cockle collected from the East Java Coast, in order to assess public health risks. The present study revealed that cockle collected along East Java Coast demonstrated a different level of metal concentrations in different sampling locations. Cockles from GR contained the highest Cd, Zn, and Hg, cockles from SU presented the highest Pb, and cockles from PA contained the highest Cu and Cr. Cockles from MU contain the lowest Pb and Zn; meanwhile, the lowest levels of Cd, Hg, Cu, and Cr are found in cockles from PR. The different levels of metals in cockles could be influenced by many factors such as sources of heavy metals, distance from estuaries (mouth of river), and oceanographic condition. Sampling locations LA, GR, BA, SU, SI, and PA are close to or near the estuaries of the rivers; therefore, cockles from these sites contain metals relatively higher than cockles from MU and PR which are far from the estuaries.

The metal concentration in the tissues of cockles was in the order Zn > Cu > Cr > Hg > Cd > Pb. Zinc is an essential micronutrient and is involved in a number of physiological functions such as protein synthesis and energy metabolism for both animals and humans. As an essential constituent of many enzymes, therefore, a relatively high level of zinc is maintained in the body of many organisms, including cockle [26]. The level of zinc in cockles of the present study was lower than the zinc level in cockles from Kuala Juru, Kuala Karau, and Jeram, Malaysia [27]; Newcastle, NSW, Australia [28]; and South Island of New Zealand [29]. However, our results were higher than those in cockles from Penang, Malaysia [30], and relatively comparable with cockles from Seine Estuary, Europe [8]; Moroccan Atlantic lagoons [31]; and Ria de Aveiro, Portugal [32] (Table 4). None of the cockles from the East Java Coast contain Zn that exceeds the permissible limit for human consumption, according to national and international standards (Table 5). Zinc is an essential element in our diet, but an overdose of zinc may lead to electrolyte imbalance, nausea, anemia, and lethargy [33, 34]. Zinc is also used for the treatment of diabetes, Down syndrome, Alzheimer's disease, and peptic ulcers [35]. Further, zinc deficiency resulting from poor diet, alcoholism, and malabsorption causes dwarfism, hypogonadism, and dermatitis [34].

A comparison with data in the literature showed that the levels of Cd in tissues of cockles in this study were relatively similar to those of cockles from other regions in the world (Table 4). The level of Cd in cockles from all sites of the East Java Coast exceeds the permissible limit for human consumption, according to EC [20] (Table 5). Cd is a highly toxic metal and can accumulate in the liver and kidney of mammals through the food chain [36]. It also produces shock and acute renal failure and affects the central nervous system of children [37].

Compared with other findings, the Pb levels in tissues of cockles of our study were lower than Pb levels in cockles from Asajaya, Sarawak, Malaysia [38], and Moroccan Atlantic lagoons [31], but their levels were relatively similar than those from Seine Estuary, Europe [8]; Ria de Aveiro, Portugal [32]; and Newcastle, NSW, Australia [28]. The levels of Pb of the present study were higher than our previous findings [13], especially in the sampling sites located in the northern coast of East Java. This fact indicates that the water runoff from upstream areas which contain Pb increases considerably. Cockles from certain sites of the East Java Coast contained Pb that exceeds the permissible limit for human consumption of FAO [39] and EC [20] (Table 5). Overconsumption of Pb can cause renal failure and liver damage in humans [40], affect the immune system, and disturb nervous system development in young children [41]. Moreover, lead is also accumulated in teeth, bone, lung, spleen, and brain, and it goes through the blood-brain barrier and the placenta [41].

The concentrations of Hg found in cockles of the East Java Coast (0.04–0.06 *μ*g kg^−1^) were comparable with those from Penang, Malaysia [30] (Table 4). Only cockle from Gresik presented Hg levels higher than the maximum permissible levels of seafood set by national and international standards (Table 5). Continuous exposure and consumption of large quantities of this cockle can pose a potential health hazard. Mercury can affect the central nervous system and cause neurodegenerative diseases and renal and immunological problems in humans [42, 43].

A comparison with data in the literature (Table 4) showed that Cu levels in cockles in our study were lower than the values found in cockles from South Island of New Zealand [29]. The levels of Cu in cockles from East Java Coast were below the toxic limit for human consumption according to national and international standards (Table 5). Cu is required for several enzymatic reactions and is necessary for the synthesis of hemoglobin [44], but it causes adverse health problems if the intake of Cu is high. Excess accumulation of copper has been reported to cause liver diseases [45], dermatitis, and neurological disorders [46]. Otherwise, copper deficiency in humans can cause bone demineralization, depressed growth, depigmentation, and gastrointestinal disturbances [46]. Consuming this cockle in adequate amount, therefore, can prevent Cu deficiency because they contain relatively high levels of Cu.

The concentrations of Cr in cockles from the East Java Coast were higher than those recorded in cockles from Penang, Malaysia [30], and Ria de Aveiro, Portugal [32]. The levels of Cr in cockles from all location of East Java Coast were higher than the Hong Kong standard (Table 5). Chromium is an essential element for the insulin molecule to bring glucose into the cells for glycolysis. However, long-term exposure to chromium can cause damage to the nose, skin, and lungs, stomach upsets and ulcers, convulsions, and even death [47].

Cockles are an important source of cheap protein for fishing communities in the East Java Coast. This fishing community is potentially exposed to high levels of metals, especially when they consume large amounts of cockles. To evaluate whether metal levels found in cockles are safe for consumers, the calculation of estimated weekly intake (EWI) of metals should be conducted.  Table 2 represents the EWI of Cd, Pb, Zn, Hg, Cu, and Cr for humans (calculated based on adult people with 60 kg body weight). The EWIs of Pb, Zn, and Cu of all locations were below the recommended values of PTWI (Table 2). The EWIs of Cr of all locations were higher than the PTWI value; meanwhile, only the EWIs of Cd and Hg of Gresik were higher than the PTWI value (Table 2). Additionally, this study showed that the THQ values for Cd at Gresik; THQs for Hg at Gresik, Surabaya, and Pasuruan; and THQs for Cr at all locations exceeded one; therefore, these metals pose a potential health risk for consumers especially their noncarcinogenic effects (e.g., reproductive toxicity, teratogenicity, or liver toxicity) over lifetime exposure [3]. It can be recommended that to minimize the health risks due to the negative effect of Cd, Hg, and Cr, it is necessary to reduce the consumption of cockles.

## 5. Conclusions

This present study provides the valuable data for trace metals in cockles living in the East Java Coast. The concentrations of Cd, Pb, Hg, and Cr in cockles in certain location of East Java Coast were found to be higher than the permissible limit for human consumption, according to Indonesia and international standards. The EWIs of Cd, Hg, and Cr for coastal people in certain location of East Java Coast were higher than the PTWI. In addition, the THQs of Cd, Hg, and Cr were also higher than one indicating these metals pose a potential health risk for consumers. According to this study, it is recommended to reduce the consumption of these bivalves in order to minimize the serious effects of metals to human health. A monitoring program (such as measuring regularly the levels of metals in seafood including cockles) should also be conducted in order to anticipate the health risk.

## Figures and Tables

**Table 1:** Concentrations of metals in cockles from East Java Coast.

Location	*N*	Metal (mg kg^−1^)					
		Cd	Pb	Zn	Hg	Cu	Cr
Lamongan	6	0.37 ± 0.09^bc^	0.44 ± 0.09^bc^	16.64 ± 1.02^b^	0.13 ± 0.01^a^	2.62 ± 0.32^ab^	1.93 ± 0.31^ab^
Gresik	6	0.82 ± 0.12^d^	0.37 ± 0.13^bc^	19.04 ± 1.00^b^	1.47 ± 0.30^b^	4.11 ± 0.35^d^	3.06 ± 0.37^bc^
Bangkalan	6	0.34 ± 0.05^bc^	0.43 ± 0.11^bc^	10.69 ± 1.82^a^	0.02 ± 0.01^a^	2.07 ± 0.15^ab^	1.69 ± 0.37^ab^
Surabaya	6	0.32 ± 0.08^bc^	0.54 ± 0.11^c^	12.88 ± 1.71^a^	0.15 ± 0.04^a^	4.01 ± 0.45^cd^	2.50 ± 0.76^abc^
Sidoarjo	6	0.39 ± 0.06^bc^	0.44 ± 0.05^bc^	12.19 ± 2.48^a^	0.12 ± 0.02^a^	2.99 ± 0.57^bc^	2.47 ± 0.83^abc^
Pasuruan	6	0.42 ± 0.07^c^	0.51 ± 0.29^c^	11.60 ± 2.87^a^	0.15 ± 0.01^a^	4.76 ± 1.17^d^	3.79 ± 1.62^c^
Muncar	6	0.27 ± 0.04^b^	0.10 ± 0.08^a^	10.22 ± 1.80^a^	0.12 ± 0.01^a^	2.57 ± 0.41^ab^	2.08 ± 0.26^ab^
Prigi	6	0.11 ± 0.05^a^	0.25 ± 0.06^ab^	10.38 ± 0.77^a^	0.02 ± 0.01^a^	1.79 ± 0.37^a^	1.64 ± 0.45^a^

Note: lowercase letters indicate significant differences (*p* < 0.05, a < b < c < d), *N* = number of replication.

**Table 2:** The estimated weekly intake (EWI) of metals by consuming cockles.

Estimated weekly intake (EWI) of metal (*μ*g kg^−1^ BW) by location	Metal					
	Cd	Pb	Zn	Hg	Cu	Cr
Lamongan	6.06	7.23	271.87	2.18	42.90	31.63
Gresik	13.29	6.06	311.06	24.06	67.13	49.97
Bangkalan	5.55	7.07	174.63	0.34	33.81	27.59
Surabaya	5.21	8.74	210.46	2.52	65.45	40.72
Sidoarjo	6.40	7.07	199.02	2.02	48.79	40.38
Pasuruan	6.90	8.42	189.60	2.52	77.72	61.91
Muncar	4.54	1.68	167.06	2.02	42.05	33.98
Prigi	1.85	4.20	169.92	0.34	29.27	26.75
The PTWI standard of metal (*μ*g kg^−1^ BW)	7	25	7000	5.6	3500	15
References	EC [20]	EC [20]	WHO [21]	WHO [22]	WHO [21]	USEPA [23]

**Table 3:** Target hazard quotient (THQ) estimate for analyzed metals evaluated in cockles from East Java Coast.

THQ	Metal					
	Cd	Pb	Zn	Hg	Cu	Cr
Lamongan	0.86	0.27	0.09	0.86	0.16	1.81
Gresik	1.91	0.25	0.15	11.43	0.24	2.38
Bangkalan	0.79	0.29	0.08	0.16	0.12	1.31
Surabaya	0.75	0.36	0.10	1.17	0.23	1.94
Sidoarjo	0.91	0.29	0.09	0.93	0.17	1.92
Pasuruan	0.91	0.32	0.09	1.17	0.26	2.78
Muncar	0.63	0.07	0.08	0.93	0.15	1.62
Prigi	0.26	0.17	0.08	0.16	0.10	1.28
Oral reference dose (RfD, *μ*g kg^−1^ BW day^−1^)	1	3.5	300	0.3	40	3

**Table 4:** Concentrations of metals (mg kg^−1^ ww) in cockle from different areas of the world.

Location	Species of cockle	Cd	Pb	Zn	Hg	Cu	Cr	Reference
Seine Estuary, Europe	*Cerastoderma edule* (Linnaeus, 1758)	0.07-0.40	<0.02-0.72	16.2-31.0	—	0.84-4.58	—	[8]
Moroccan Atlantic lagoons^∗^	*Cerastoderma edule*	<0.001-0.82	1.67-7.77	7.9-31.1	—	1.01-7.75	—	[31]
Kuala Juru, Kuala Karau, and Jeram, Malaysia^∗^	*Anadara granosa*	0.27-1.95	—	18.93-41.91	—	1.11-1.52	—	[27]
Penang, Malaysia	*Anadara granosa*	0.79-0.93	0.11-0.14	0.17-0.29	1.21-1.50	0.15-23	0.15-0.19	[30]
Eastern region of Java Sea	*Anadara granosa*	0.2-0.4	<0.04	—	<0.01-0.05	—	—	[13]
South Island of New Zealand	*Austrovenus stutchburyi* (Wood, 1828)	0.2-0.45	—	45-65	—	6-10	—	[29]
Ria de Aveiro, Portugal	*Cerastoderma edule*	0.06-0.16	1-5	35-55	—	2-5	0.3-0.9	[32]
Asajaya, Sarawak, Malaysia^∗^	*Anadara granosa*	0.28-0.45	0.55-0.90	—	—	—	—	[38]
Newcastle, New South Wales, Australia^∗^	*Anadara trapezia* (Deshayes, 1839)	0.2-3.5	0.1-4.5	16.5-37.1	—	0.8-3.5	—	[28]
Along East Java Coast	*Anadara granosa*	0.11-0.82	0.10-0.54	10.22-19.04	0.02-1.47	1.79-4.76	1.64-3.79	Present study

^∗^Data in dry weights were transformed to wet weight by dividing by a factor 4.856.

**Table 5:** Maximum permissible limits of trace metals in seafood (mg kg^−1^ ww) according to national and international guidelines.

Standard	Cd	Pb	Zn	Hg	Cu	Cr
WHO [48]	1	2	100	—	30	50
FAO [39]	0.5	0.5	40	—	30	—
USEPA [49]	2	4	120	—	120	8
EC [20]	0.05	0.2	—	0.5	—	—
Hong Kong [50, 51]	2	6	—	0.5	—	1
Australia [52]	2	2.5	—	0.5	—	—
Indonesia [13]	1	2	100	1	20	—

## Data Availability

The data used to support the findings of this study are available from the corresponding author upon request.
